# Intrauterine thrombosis of umbilical artery - case report: Trombose intrauterina de artéria umbilical - relato de caso

**DOI:** 10.1590/1516-3180.2016.00081203

**Published:** 2016-06-03

**Authors:** Gustavo Henrique de Oliveira, Cristiane de Moraes Dias, Denise Cristina Mós Vaz-Oliani, Antonio Hélio Oliani

**Affiliations:** I MD, MSc. Visiting Professor, Interdepartmental Centre for Fetal Medicine, Faculdade de Medicina de São José do Rio Preto (FAMERP), and Attending Physician, Instituto de Medicina Reprodutiva e Fetal SS (IMR), São José do Rio Preto, SP, Brazil.; II MD. Member of the Interdepartmental Centre for Fetal Medicine, Faculdade de Medicina de São José do Rio Preto (FAMERP), and Attending Physician, Instituto de Medicina Reprodutiva e Fetal SS (IMR), São José do Rio Preto, SP, Brazil.; III MD, MSc, PhD. Coordinator, Centre for Fetal Medicine, Faculdade de Medicina de São José do Rio Preto (FAMERP), and Adjunct Professor, Department of Gynecology and Obstetrics, São José do Rio Preto, SP, Brazil.; IV MD, MSc, PhD. Head, Department of Gynecology and Obstetrics, Faculdade de Medicina de São José do Rio Preto (FAMERP), and Technical Director, Instituto de Medicina Reprodutiva e Fetal SS (IMR), São José do Rio Preto, SP, Brazil.

**Keywords:** Thrombosis, Prenatal diagnosis, Fetal growth retardation, Ultrasonography, prenatal, Embryonic and fetal development, Trombose, Diagnóstico pré-natal, Retardo do crescimento fetal, Ultrassonografia pré-natal, Desenvolvimento embrionário e fetal

## Abstract

**CONTEXT:**

Umbilical cord thrombosis is related to greater fetal and perinatal morbidity and mortality. It is usually associated with umbilical cord abnormalities that lead to mechanical compression with consequent vascular ectasia. Its correct diagnosis and clinical management remains a challenge that has not yet been resolved.

**CASE REPORT:**

This study reports a case of umbilical artery thrombosis that occurred in the second half of a pregnancy. The umbilical cord was long, thin and overly twisted and the fetus presented severe intrauterine growth restriction. The clinical and histopathological findings from this case are described.

**CONCLUSIONS:**

This case report emphasizes the difficulty in diagnosing and clinically managing abnormalities of intrauterine life with a high chance of perinatal complications.

## RESUMO

**CONTEXTO:**

A trombose do cordão umbilical está relacionada com o aumento da morbimortalidade fetal e perinatal. É geralmente associada a alterações do cordão umbilical que levam à compressão mecânica com consequente ectasia vascular. Seu correto diagnóstico e manejo clínico é um desafio que não está ainda bem esclarecido.

**RELATO DE CASO:**

Neste relato se descreve caso de trombose da artéria umbilical de ocorrência na segunda metade da gravidez associada a cordão umbilical longo, fino, excessivamente retorcido, associado a feto com restrição de crescimento intrauterino grave. São descritos seus achados clínicos e histopatológicos correlacionados.

**CONCLUSÃO:**

Este relato de caso reforça a dificuldade diagnóstica e de manejo clínico em alteração da vida intrauterina com grande possibilidade de complicações perinatais.

## INTRODUCTION

Vascular thrombosis of the umbilical cord has been described as an abnormality that increases fetal morbidity and mortality during intrauterine life and the perinatal period. Its incidence is estimated to be one case in every 1300 gestations and up to one in every 250 deliveries when only high-risk pregnancies are taken into consideration.^1^ The main causes of this phenomenon are abnormalities of the umbilical cord, such as: excessive twisting, presence of a true knot, very long or very short cord, loops around the body or cervical region, marginal or velamentous placental insertion and very thin cord with little Wharton jelly. Such situations may lead to vascular ectasia followed by thrombosis with a high risk of impairment of fetal wellbeing.^2^^,^^3^ In this article, we report a case of umbilical artery thrombosis that was diagnosed during the prenatal period with development of significant intrauterine growth restriction.

## CASE REPORT

The patient was 30 years of age, in her second pregnancy, without any relevant personal or family history, and had been attending prenatal care for low-risk pregnancies. Her previous pregnancy had run its course without any complications, with a neonate born at term with adequate weight.

At 32 weeks of gestation in the second pregnancy, the uterine height was observed in a routine consultation to be less than what would be expected for the gestational age. The patient was referred for ultrasonography, from which the fetal weight was diagnosed as below the 5^th^ percentile for the gestational age, with a single umbilical artery and presence of a cervical umbilical cord loop. Doppler flow analysis did not show any abnormalities in this examination.

**Figure 1.**
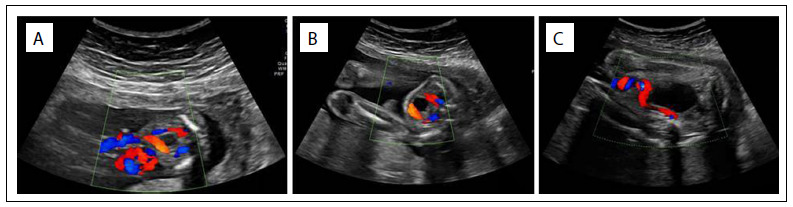
Imaging of the case at 18 weeks (1a) and 22 weeks (1b), and then at 32 weeks (1c), when the “disappearance” of one of the umbilical arteries was noticed.

Retrospective analysis on ultrasound imaging on the patient that had been produced during this pregnancy showed that there were no abnormalities of growth, fetal structure or umbilical cord up to the 22^nd^ week (Figures 1A, B and C). The hypothesis of spontaneous intrauterine umbilical artery thrombosis was raised. Weekly serial ultrasound imaging was started and fetal pulmonary maturation treatment was instituted using corticosteroids.

**Figure 2.**
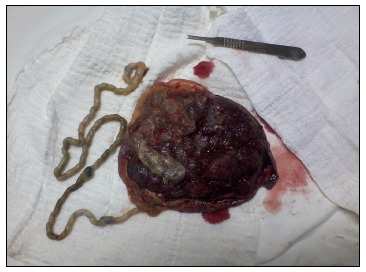
Imaging in which the long, thin, excessively twisted umbilical cord was identified.

At 34 weeks of gestation, cessation of fetal growth was observed and also a slight decrease in amniotic fluid volume. Doppler velocimetry on the umbilical artery showed that the pulsatility index value was close to the 90^th^ percentile for the gestational age, with adequate venous flows. It was decided to implement early delivery by means of caesarean section. It was observed during the procedure that the amniotic fluid had the appearance of meconium and that the umbilical cord was thin and approximately 80 cm in length, with excessive twisting (Figure 2) and two tight cervical loops.

The newborn was male, with first-minute Apgar of 9 and fifth-minute Apgar of 10, weighing 1080 g. His gestational age from the somatic Capurro method was 34 weeks. He was referred to the neonate intensive care unit only because of low weight. He presented good postnatal evolution and was discharged from hospital on the 37^th^ day of life. The histological analysis showed massive thrombosis in one of the umbilical arteries.

## DISCUSSION

Over recent decades, there have been great advances in prenatal care, especially through the evolution of ultrasound imaging and better understanding of embryology and fetal life. Concomitantly, pregnant women have become more demanding, such that they require their obstetrician and prenatal doctor (who are often faced with threats and lawsuits) to provide safe and precise follow-up.^4^ However, situations without any precise diagnosis or clear management are frequently encountered. It is known that umbilical cord thrombosis is associated with adverse fetal and perinatal outcomes, but its diagnosis and follow-up constitutes a clinical challenge.

Umbilical cord abnormalities such as an overly long cord (more than 70 cm) or short cord (less than 35 cm), excessive twisting (more than 0.3 cm/loop, reduced diameter (less than 8.0 mm), anomalous placental insertions and presence of true knots and loops are well-established risk factors for gestational complications and impairment of fetal wellbeing. These abnormalities lead to mechanical compression or blood ectasia in the fetal vascular path.^5^^,^^6^^,^^7^^,^^8^^,^^9^^,^^10^ Moreover, such abnormalities are related to the etiology of umbilical cord thrombosis, and the risk of their occurrence is generally three times higher in the presence of cord thrombosis.^11^ In a study on autopsies conducted on 139 fetuses after spontaneous intrauterine death, vascular thrombosis of the umbilical cord was identified in 20% of the cases, mainly when there was excessive twisting of the umbilical cord and reduction of its diameter.^6^ In situations of presence of umbilical cord abnormalities, intensive fetal monitoring during delivery and histological analysis on the umbilical cord are recommended, especially in cases of intrauterine death.^12^

A few studies on umbilical cord thrombosis have already been conducted. The systematized results from the main database in the literature are presented in Table 1. There seems to be slight male predominance. Venous thrombosis alone is the most common occurrence, found in approximately 70% of the cases, followed by concomitant arterial and venous thrombosis in 20% of the cases and arterial thrombosis alone in 10% of the cases.^1^ It is believed that the incidence of umbilical artery thrombosis alone is very small, ranging from 0.0025% to 0.045% of gestations.^1^^,^^3^ Although venous thrombosis is more frequent, it is believed that adverse outcomes are more common in arterial thrombosis.^1^ In the main published papers on this subject, associations with growth restriction, fetal death, meconium in the amniotic fluid, acute fetal distress during labor and higher rates of emergency caesarian sections have been noted.^2^^,^^3^^,^^6^^,^^13^ The most common abnormalities of the umbilical cord associated with umbilical artery thrombosis are: short or long cord, excessive twisting and anomalous placental insertions.^3^^,^^11^

**Table 1.**
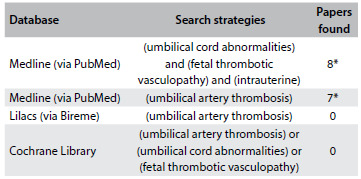
Search of the literature in medical databases, for cases of vascular thrombosis of the umbilical cord and prenatal diagnosis

*Two studies were available through both the first and the second database searches.

## CONCLUSIONS

In the case presented here, which occurred in a low-risk pregnancy in which up to the 22^nd^ week there had been no suspicion of abnormality, spontaneous intrauterine umbilical artery thrombosis was detected in the third trimester of pregnancy and was associated with a thin and long umbilical cord, with excessive twisting and cervical loops. Severe intrauterine growth restriction and deterioration of fetal wellbeing were also observed. The diagnosis only became possible through making comparisons with the patient’s initial ultrasound screenings, in which both umbilical arteries were clearly identified, with subsequent confirmation through histological analysis. Unfortunately, findings such as cord length abnormality and cord twisting are not commonly identified. In the presence of umbilical cord abnormalities, the risk of complications during intrauterine life, at the time of delivery and during the perinatal period, needs to be taken into consideration. Although the procedure in such cases is not well established, regular monitoring of fetal wellbeing, implementation of pulmonary maturation and early delivery in the event of fetal deterioration are recommended. Fetal monitoring is important because of the risk of adverse outcomes.

## References

[1] Heifetz SA (1988). Thrombosis of the umbilical cord: analysis of 52 cases and literature review. Pediatr Pathol.

[2] Tantbirojn P, Saleemuddin A, Sirois K (2009). Gross abnormalities of the umbilical cord: related placental histology and clinical significance. Placenta.

[3] Sato Y, Benirschke K (2006). Umbilical arterial thrombosis with vascular wall necrosis: clinicopathologic findings of 11 cases. Placenta.

[4] MacLennan A, Nelson KB, Hankins G, Speer M (2005). Who will deliver our grandchildren? Implications of cerebral palsy litigation. JAMA.

[5] Avagliano L, Marconi AM, Candiani M, Barbera A, Bulfamante G (2010). Thrombosis of the umbilical vessels revisited. An observational study of 317 consecutive autopsies at a single institution. Hum Pathol.

[6] Peng HQ, Levitin-Smith M, Rochelson B, Kahn E (2006). Umbilical cord stricture and overcoiling are common causes of fetal demise. Pediatr Dev Pathol.

[7] Baergen RN (2007). Cord abnormalities, structural lesions, and cord “accidents”. Semin Diagn Pathol.

[8] Chan JS, Baergen RN (2012). Gross umbilical cord complications are associated with placental lesions of circulatory stasis and fetal hypoxia. Pediatr Dev Pathol.

[9] Machin GA, Ackerman J, Gilbert-Barness E (2000). Abnormal umbilical cord coiling is associated with adverse perinatal outcomes. Pediatr Dev Pathol.

[10] Baergen RN, Malicki D, Behling C, Benirschke K (2001). Morbidity, mortality, and placental pathology in excessively long umbilical cords: retrospective study. Pediatr Dev Pathol.

[11] Redline RW (2004). Clinical and pathological umbilical cord abnormalities in fetal thrombotic vasculopathy. Hum Pathol.

[12] Hasegawa J, Matsuoka R, Ichizuka K, Sekizawa A, Okai T (2009). Ultrasound diagnosis and management of umbilical cord abnormalities. Taiwan J Obstet Gynecol.

[13] Klaritsch P, Haeusler M, Karpf E, Schlembach D, Lang U (2008). Spontaneous intrauterine umbilical artery thrombosis leading to severe fetal growth restriction. Placenta.

